# Genetic Code-Locking Confers Stable Virus Resistance to a Recoded Organism

**DOI:** 10.1021/acs.biochem.5c00075

**Published:** 2025-07-01

**Authors:** Jérôme F. Zürcher, Alexandre Dickson, Tomás Kappes, Askar A. Kleefeldt, Kim C. Liu, George P. C. Salmond, Jason W. Chin

**Affiliations:** † Medical Research Council Laboratory of Molecular Biology, Francis Crick Avenue, Cambridge CB2 0QH, England, U.K.; ‡ Department of Biochemistry, 2152 University of Cambridge, Tennis Court Road, Cambridge CB2 1GA, England, U.K.

## Abstract

The genetic code defines the correspondence between codons in genes and amino acids in proteins. Reassignment of sense codons to different amino acids can create cells with refactored genetic codes that are distinct from the canonical genetic code. By encoding essential genes according to the refactored genetic code, this code becomes locked-in, making it essential to the host cell. Here, we show that refactoring the structure of the genetic code alone is sufficient to confer temporary resistance to complex mobile genetic elements, such as viruses. However, when the refactored genetic code is not locked-in, it can revert, leading to loss of resistance. Thus, locking the refactored genetic code may be crucial for maintaining stable, long-term resistance in the face of sporadic and unpredictable viral infection.

## Graphical abstract

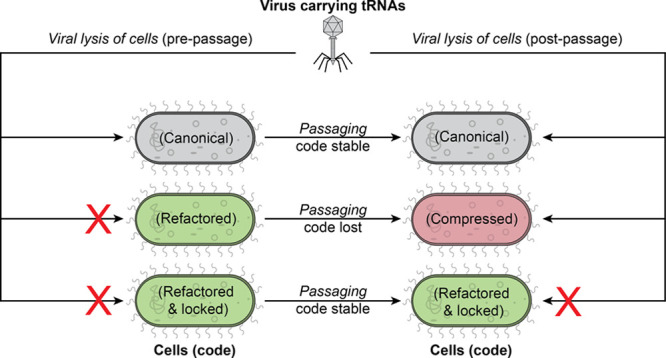

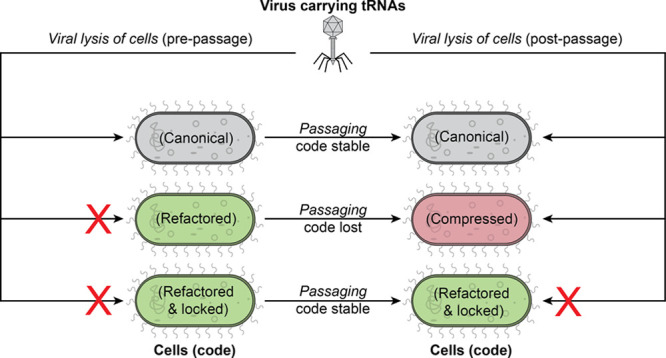

## Introduction

The genetic code defines the set of rules by which the information stored in nucleic acids is translated into proteins.^1,2^ Due to the near-universality of the genetic code, genetic information within coding sequences from almost any organism can be read and translated in a different host organism. The sharing of innovation across the tree of life is a major driver of evolution; however, it also allows viruses and other detrimental genetic elements to hijack cells and replicate at the cell’s expense.^3–6^ Natural deviations from the standard genetic code may protect cells from detrimental genetic elements that use the standard genetic code. Furthermore, altering the genetic code of a target organism offers a unique and potentially universal strategy to confer virus resistance.^7–10^ Organisms with long-term resistance to viral infection have the potential to be of value in research and biomanufacturing.^11^

Genome synthesis and editing provide an opportunity to change the genetic code of organisms and endow them with favorable properties.^12–15^ We previously reported a strain of with a synthetic genome where all annotated instances of two serine codons (TCG, TCA) were replaced with synonyms, and the amber stop codon (TAG) was replaced with TAA.^13^ This organism-named Syn61-only uses 61 codons to encode its proteome. Further, we deleted the genes (*serT*, *serU*, and *prfA*) that specify the decoders (tRNAs and Release factor 1) of TCG, TCA, and TAG codons (Figure 1a). This gave rise to Syn61Δ3, a codon compressed organism that is no longer able to decode TCG, TCA, and TAG codons; this organism does not have the ability to read a subset of codons present in the canonical genetic code and therefore cannot translate the genes of mobile genetic elements that use a standard genetic code.^7^ Consequently, this organism is resistant to invasion by genetic elements that contain TCG, TCA, and TAG codons in their genes (Figure 1b). We previously showed that Syn61Δ3 is resistant to infection by a broad range of viruses and transfer of a conjugative mobile genetic element (F plasmid) that encodes its genes according to the canonical genetic code [F (WT)].^7,8^ However, mobile genetic elements that encode their own tRNAs [F (WT + *serT*)], and natural phage that carry seryl-tRNAs, rescue the ability to read TCG and TCA and can be propagated in Syn61Δ3 (Figure 1c).^8,9^

**Figure 1:**
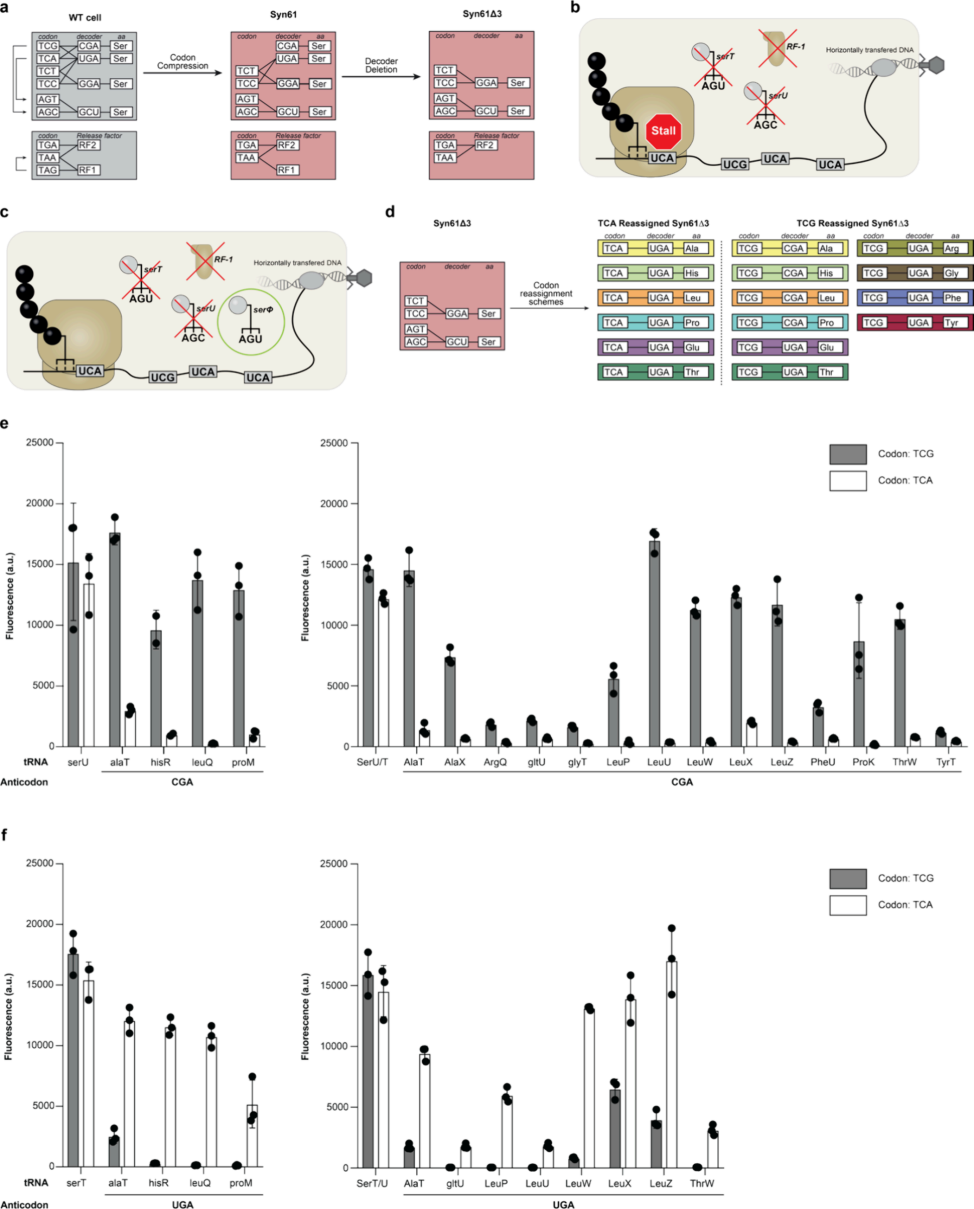
Refactoring the genetic code. **a**. Codon compression through whole genome synthesis followed by tRNA deletion gave rise to Syn61Δ3 a strain where two serine codons (TCG and TCA) and the amber stop codon (TAG) are unassigned. **b**. A virus invades Syn61Δ3. In this cell, serU (encoding tRNA^Ser^_CGA_), serT (encoding tRNA^Ser^_UGA_, and prfA (encoding RF-1) were deleted making TCG, TCA, and TAG codons unreadable. The ribosome will stall at these codons on an mRNA that contains them. As the viral mRNA contains these codons the viral genes cannot be translated, and the virus cannot propagate. **c**. A virus encoding its own tRNA (serΦ) invades Syn61Δ3; a cell where TCG, TCA, and TAG are not decoded. The tRNA expressed from the viral genome rescues the ability to translate TCG and TCA codons. Therefore, viral genes can be expressed in the cell and the virus can propagate. **d**. Codons were reassigned to natural amino acids distinct from serine through engineering of endogenous tRNAs. TCA was reassigned to Alanine, Histidine, Leucine, Proline, Glutamate, or Threonine. TCG was reassigned to Alanine, Histidine, Leucine, Proline, Glutamate, Threonine, Arginine, Glycine, Phenylalanine, or Tyrosine. Target codons are indicated to the left; anticodon and amino acid (aa) of the corresponding decoders to the right. **e**. Anticodon (CGA) modified tRNAs suitable for genetic code refactoring. The translational activity of these tRNAs was assessed through suppression of a TCG or TCA codon at position 3 of a sfGFP gene in Syn61Δ3. Measured fluorescence serves as a read-out for translational activity of a given tRNA on a given codon. tRNAs suitable for genetic code refactoring show substantial activity on their cognate codon and minimal activity on the off-target codon. Independent replicates shown as individual dots (*n* = 3 [*n* = 2 for hisR], bars indicate standard deviation). Data in left panel is reproduced from prior work (values were adjusted for path length).^8^ **f**. Anticodon (UGA) modified tRNAs suitable for genetic code refactoring. The translational activity of these tRNAs was assessed through suppression of a TCG or TCA codon at position 3 of a sfGFP gene in Syn61Δ3. Measured fluorescence serves as a read-out for translational activity of a given tRNA on a given codon. tRNAs suitable for genetic code refactoring show substantial activity on their cognate codon and minimal activity on the off-target codon. Independent replicates shown as individual dots (*n* = 3, bars indicate standard deviation). Data in left panel is reproduced from prior work (values were adjusted for path length).^8^

Refactoring the genetic code through reassignment of TCG and TCA to amino acids distinct from serine can create cells with genetic codes distinct from the canonical genetic code [e.g., Syn61Δ3 (tRNA^Ala^_CGA_, tRNA^His^_UGA_), Syn61Δ3 (tRNA^Leu^_CGA_, tRNA^Leu^_UGA_)]. We previously reported the independent reassignment of TCG and TCA codons to four distinct amino acids enabling the creation of 16 unique refactored genetic codes. Cells with refactored genetic codes show increased resistance to invasion by mobile genetic elements carrying their own tRNAs and enable the containment of genetic information within a specific host cell.^8,9^ We showed that writing genes that are essential for cell survival in a refactored code–referred to as code-locking–was necessary to confer to cells the resistance to a conjugative element carrying a seryl-tRNA. We also showed that code locked cells conferred resistance to phage carrying a seryl-tRNA.^8^ Genetic code refactoring also allows generation of multiple bacterial populations that are genetically isolated from one another; each population can only interpret correctly the genetic information written in its specific refactored genetic code but not information written in the canonical genetic or a different refactored code.^8^ This feature provides a blueprint for containment of genetic information in the chosen refactored strain to prevent it from being transferred to organisms in the wild.

Here we screen anticodon modified versions of all endogenous tRNAs for their ability to decode TCG and TCA codons. We find 6 additional reassignments for TCG and 2 additional reassignments for TCA, expanding the number of unique, engineerable refactored genetic codes in Syn61Δ3 from 16 to 60 (Figure 1d). We show that while complete resistance to invasion by conjugative genetic elements carrying their own tRNA requires code-locking,^8^ genetic code refactoring without code locking is sufficient to confer resistance to phage carrying their own tRNA. However, we show that code-locking in an organism is crucial for its sustained resistance to viruses carrying their own tRNAs. In organisms without code-locking, the refactored genetic code is nonessential and can be reverted to a compressed code; this leads to loss of resistance to viruses carrying their own tRNAs upon passaging. In organisms with locked refactored codes, refactoring confers an advantage to the organism and these cells maintain broad resistance to phage infection after passaging.

## Materials and Methods

### Strains and Plasmids Used in This Study

We used Syn61 ev2 (Syn61WT) and Syn61Δ3 ev4 (Syn61Δ3) for codon reassignment and phage resistance experiments. For plasmid cloning we used Dh10b and Syn61ev2.

We used the following plasmids in this study – pSC101 (tRNA plasmids), pMB1 (code-locking spectinomycin plasmids), and pBAD (sfGFP-His6 plasmids). For a full list of plasmids see Supplementary Table 4.

### tRNA Plasmid Construction

We constructed pSC101 based tRNA plasmids by Gibson assembly (HiFi assembly NEB) of multiple fragments. The backbone fragment was generated by PCR. tRNA genes were ordered as synthetic DNA (gBLOCKS IDT).

### Translational Assays

sfGFP-His_6_ genes bearing a single TCG or TCA codon at position 3 were expressed in Syn61Δ3 cells harboring a pSC101 plasmid encoding a tRNA gene. Syn61Δ3 cells containing a pBAD_sfGFP reporter plasmid were grown from glycerol stocks in 5 mL of prewarmed 2xYT media containing 50 μg/mL apramycin overnight at 37 °C while shaking at 220 rpm. From this overnight culture, electrocompetent cells were prepared. pSC101-based tRNA plasmids were electroporated into the Syn61Δ3 electrocompetent cells. Cells were recovered in 96-well plates for 90 min in 500 μL SOC. Subsequently, a tenth of the recovered culture (50 μL) was inoculated in 450 μL of prewarmed 2xYT media supplemented with 200 μg/mL hygromycin and 50 μg/mL apramycin. After recovering for 24–36 h, 37 °C, 750 rpm, expressions were setup in 96-well microtiter plate format. Overnight cultures were inoculated 1:50 into 500 μL of prewarmed 2xYT containing hygromycin (200 ng/μL), apramycin (50 ng/μL), and l-arabinose (0.2%) and incubated for 20–24 h at 37 °C while shaking at 750 rpm. Cells were harvested by centrifugation at 3200 g for 10 min. Supernatant was discarded and cell pellets resuspended in 150 μL of PBS, 100 μL of which were transferred into a Greiner clear 96-well flat-bottom plate. In this plate OD_600_ and GFP fluorescence (λ_ex_: 485 nm; λ_em_: 520 nm) measurements were recorded on a PHERAstar FS plate reader (BMG LABTECH) (gain setting of 0, focal adjustment of 0 mm, with path length correction).

Data reproduced from an earlier publication was measured in a different plate. To make it comparable to the data measured here we implemented a path length correction. (The data displayed in Figure 1 is equivalent to the data from an earlier publication^8^; all values were divided by a factor of 3.15 to account for path length correction).

### Purification of sfGFP-His6 Protein

Syn61Δ3 cells harboring a pSC101-based tRNA plasmid and a pBAD_sfGFP plasmid were grown for 16h in 5 mL prewarmed 2xTY media containing 200 μg/mL hygromycin, 50 μg/mL apramycin, and 0.2% l-arabinose at 37 °C while shaking at 220 rpm. Following the expression, cells were harvested by centrifugation, resuspended in 1 mL Lysis buffer (1× Bugbuster Protein Extraction Reagent (Novagen), 1× PBS, 50 μg/mL DNase 1, 20 mM imidazole, and 100 μg/mL lysozyme), and incubated at 4 °C for 1 h. The resulting lysates were centrifuged (20,000*g*) at 4 °C for 30 min to remove cell debris. The supernatant was transferred to 1.5 mL microcentrifuge tubes containing 30 μL of Ni^2+^-NTA slurry (Qiagen) and incubated for 1h at 4 °C while tumbling. Ni^2+^-NTA beads were collected by gravity filtration on a column and washed three times in 500 μL wash buffer (1× PBS, 40 mM imidazole). Protein was eluted twice with 50 μL of elution buffer (1× PBS, 300 mM imidazole, pH 8) and collected in a fresh microcentrifuge tube via centrifugation (400*g*, 4 °C, 1 min).

### Intact Protein Mass Spectrometry

ESI-MS analysis of purified sfGFP was performed using a Waters Xevo G2 mass spectrometer coupled to a modified nanoAcquity LC system. The protein samples were separated on a BEH C4 UPLC column (1.7 μm; 1.0 × 100 mm; Waters) over 20 min with a flow rate of 50 μL/min and a water/acetonitrile gradient from 2% vol/vol to 80% vol/vol. Subsequently. Eluted samples were interfaced via Zspray electrospray ionization source with the mass spectrometer (Waters). Data was acquired in positive ion mode with a range from 300 to 2000 *m*/*z* and an applied cone voltage of 30 V. Spectra were deconvoluted using the MaxEnt1 function within MassLynx software (Waters). Expected molecular weights were determined by manually editing the expected mass of wild-type sfGFP (GPMAW; Lighthouse Data) to accommodate encoded amino acid changes.

### Efficiency of Plaquing Assays

Top lawns containing 200 μL of overnight culture mixed with 4 mL of top agar were poured as an overlay on LB agar plates containing 200 μg/mL hygromycin and 75 μg/mL spectinomycin. Top lawns were dried. Subsequently, serially diluted (10-fold) phage lysates were spotted (7.5 μL per spot) on top. Plates were dried and incubated overnight at 37 °C. For concentrations where single plaques were expected full top lawns were poured to get a better assessment of plaque forming units at the given concentration (200 μL of overnight culture mixed with 10 μL of phage lysate at the concentration of interest and 4 mL of top agar poured as an overlay on LB agar plates containing 200 μg/mL hygromycin and 75 μg/mL spectinomycin). Plaque counts displayed in bar graphs stem from full top lawns. For titter lysates (>10^6^ PFU/mL) full top lawns were poured as described above to avoid lysis from without. The maximum titters used for infections with phage 06 and phage 12 were ∼7.5 × 10^9^ and ∼1.1 × 10^10^ PFU/mL respectively.

For efficiency of plaquing assays on samples from the code-reversal time-course, glycerol stocks of the given strain and given time-point were grown in 5 mL prewarmed 2xTY containing 75 μg/mL spectinomycin overnight to saturated cultures, such that these cultures stem not from a single clone but are representative of the population of cells at the given time-point.

### Phage Propagation Assays

Strains to be investigated were grown from glycerol stocks in 5 mL prewarmed 2xTY containing 75 μg/mL spectinomycin overnight to saturated cultures. In the morning these cultures were diluted (OD_600_ = 0.3) in 3 mL prewarmed 2xTY containing 75 μg/mL spectinomycin and infected with phage 12 (MOI = 0.001). As a control 3 mL prewarmed 2xTY containing 75 μg/mL spectinomycin but no cells was used. Samples were incubated for 24 h at 37 °C in a shaking incubator for phage to propagate. Then samples were diluted serially (10-fold) and spotted (7.5 μL per spot) on freshly poured and dried top lawns (200 μL of overnight culture of Syn61WT mixed with 4 mL of top agar poured as an overlay on LB agar plates containing 75 μg/mL spectinomycin) and incubated overnight at 37 °C. Plaques were counted in the spot with an appropriate dilution the next day. To calculate the PFU/mL the plaque count was multiplied with 133.3 (7.5 μL/1000 μL). The detection limit is therefore 133.3 PFU/mL.

### Phage Genome Sequencing

Phage genomic DNA was purified from 450 μL of high-titer phage lysates (∼10^10^ PFU/mL) using an established phenol/chloroform method as described previously.^16^ Then purified phage DNA was prepared for NGS using the Nextera XT DNA library preparation kit. Libraries were paired-end sequenced on a MiSeq (Illumina, reagent kit v3 (150 cycles)).

### Phage Genome Assembly and Annotation

De novo assembly of phage genomes was performed with Unicycler in short-read mode and with default options.^17^ Sequence coverage throughout the phage genome is represented as median sequencing coverage in windows of 250 bp.

### Genome Analysis

The number of TCG and TCA codons in the genomes of phage 6, phage 12 and the RK2 conjugative plasmid was determined using a custom Python 3.7 script (https://github.com/JWChin-Lab), as previously described.^7^ To determine the codon density the codon count for a given genome was simply divided by the number of kilobases in the given genome.

The assembled phage genome was annotated using multiPhATE2 as described, using the config file samples.multiPhate.config.^19^ Results from phanotateOutput.txt were processed by a custom R script before importing for viewing in Snapgene. For identification of the seryl-tRNA gene phage genomes were run through the tRNA scan webtool^20^ and seryl-tRNA hits were annotated in manually in Spangene.

### Phage Relatedness Analysis

Reference genomes T4 [NC_000866.4] and Felixounavirus LF82P2 [OZ035741.1] were obtained from Refseq (Genbank) on October third, 2024. Query coverage and percent identity (as shown Supplementary Table 1) were determined using NCBI Blast multiple sequence alignment. Phages 06, 12, and REP01–04 were aligned against T4. Phages REP05–12 were aligned against Felixounavirus LF82P2.

### Code Reversal Time-Course

Cells were grown with and without code-locking in 5 mL prewarmed 2xTY containing 75 μg/mL spectinomycin but no hygromycin. Twenty μL cells were passaged into 5 mL prewarmed 2xTY containing 75 μg/mL spectinomycin roughly every 12 h. At every passage 500 μL of dense cells were frozen as a glycerol stock at −80 °C. To assess plasmid loss, glycerol stocks from all passages were streaked onto 2xTY agar plates containing 75 μg/mL spectinomycin. 32 colonies were picked from each plate, grown to dense cultures in 96-well plates, and phenotyped for the presence of the tRNA plasmid on 2xTY agar plates containing 200 μg/mL hygromycin or 75 μg/mL spectinomycin. Clones that grow on spectinomycin but not on hygromycin were determined to have lost the tRNA plasmid.

### Pymol Structural Analysis of gp23

The structure of gp23 of T4 phage was downloaded from Uniprot [PDB identifier 6UZC]. Residues encoded by TCA were identified in the genbank file of phage 6 or phage 12 and highlighted in orange.

## Results

### Screening of Endogenous *E. coli* tRNA variants Enables Creation of Additional Refactored Genetic Codes

To expand the set of distinct refactored genetic codes, we screened anticodon modified versions of all endogenous tRNAs (excluding seryl-tRNAs and tRNAs characterized in a previous study) for their ability to decode TCG and TCA codons. We expressed a GFP gene with a TCG or TCA codon at position three of the reading frame in Syn61Δ3 cells, together with an anticodon modified tRNA and assessed translational activity by measuring GFP fluorescence (Figure 1e,f).

First, we assessed cognate decoding. We found that 22 out of 39 tRNAs with an anticodon modified to CGA show translational activity on TCG codons, and 21 out of 39 tRNAs with an anticodon modified to UGA show translational activity on TCA codons (Figure S1). For the tRNAs showing activity on cognate codons we then assessed the incorporated amino acid identity by mass-spectrometry and their noncognate activity (activity of CGA modified tRNAs on TCA; UGA modified tRNAs on TCG). We found that most anticodon modified tRNAs are still charged with the amino acid defined by the parent isoacceptor (19 out of 22 CGA modified tRNAs; 16 out of 21 UGA modified tRNAs) (Figures S1 and S2). Furthermore, most tRNAs showed high specificity for their cognate codon. However, a few tRNAs exhibited substantial translational activity on noncognate codons which could hinder specific codon reassignment (Figure S4).

tRNAs ideal for codon reassignment must (i) exclusively incorporate the correct amino acid in response to the target codon, (ii) have high translational activity at the target codon, and (iii) exhibit low activity on off-target codons to enable unique reassignment (Figure S5). Our screen identified 6 additional reassignments of TCG (arginine, glutamate, glycine, phenylalanine, threonine, and tyrosine) and 2 additional reassignments of TCA (glutamate and threonine), increasing the number of unique refactored genetic codes accessible in Syn61Δ3 to 60 (Figures 1 and S1–S4). However, most tRNAs identified for these new reassignments have substantially lower activity than previously characterized tRNAs,^8^ potentially limiting protein expression and genetic isolation.

Our screen reveals that certain isoacceptor-tRNAs are more suitable for genetic code refactoring than others. For instance, reassignment of TCA to leucine works well with UGA anticodon modified versions of *leuW*, *leuX*, and *leuZ*, but less well with a UGA anticodon modified version of *leuU* (Figures 1 and S4). Furthermore, while anticodon modified versions of *leuX* show substantial noncognate decoding, anticodon modified versions of *leuW* are highly specific to their cognate codon.

As an alternative to anticodon modified endogenous tRNAs others have used evolved leucyl-tRNAs and phage derived leucyl-tRNAs for genetic code refactoring and reported that they are more highly expressed, presumably yielding higher activity at TCR codons.^9^ However, in our hands, the translational activity of these tRNAs is not higher than that of the most active endogenous leucyl-tRNAs with modified anticodons; indeed in several cases the activity of these nonendogenous tRNAs is substantially lower than the activity of anticodon modified endogenous tRNAs (Figure S6). Additionally, we observed inconsistent growth patterns in cells expressing phage derived leucyl-tRNAs, indicating that these tRNAs may cause some toxicity.

### Codon Reassignment and Code-Locking Obstruct Horizontal Gene Transfer

Refactoring the genetic code, through reassignment of TCG and TCA codons to amino acids other than serine, created cells with genetic codes distinct from the canonical genetic code [e.g., Syn61Δ3 (tRNA^Ala^_CGA_, tRNA^His^_UGA_), Syn61Δ3 (tRNA^Leu^_CGA_, tRNA^Leu^_UGA_)]. In cells with a refactored genetic code, import of a seryl-tRNA does not simply revert the code back to WT but leads to ambiguous decoding of TCG and TCA codons (Figure 2a). This leads to stochastic amino acid misincorporation in response to TCG and TCA codons within genes that are read in the cell.

**Figure 2:**
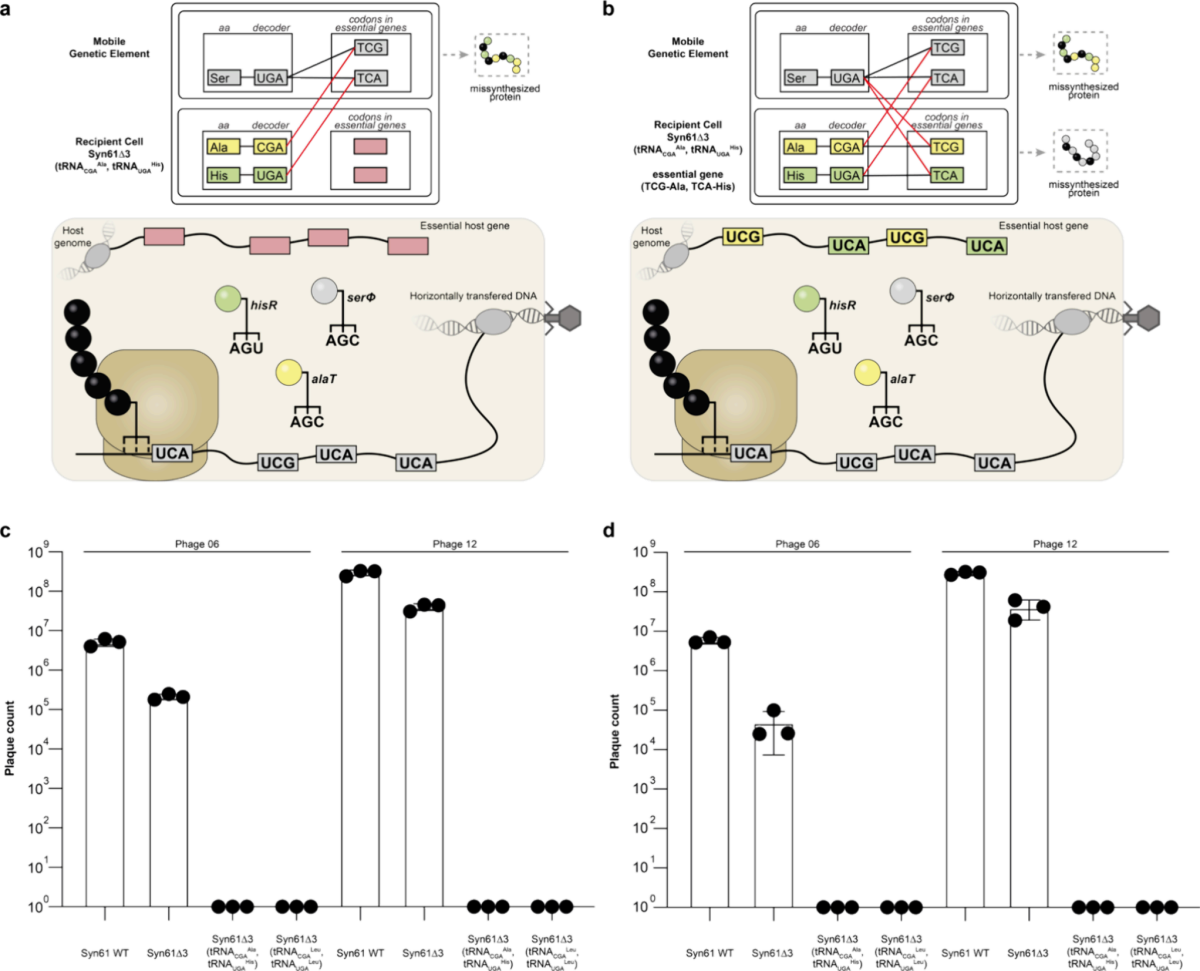
Effects of code refactoring and code-locking on phage infection. **a**. A mobile genetic element encoding its own tRNAs invades a cell with a refactored genetic code. Competition between host and invading tRNAs leads to ambiguous decoding of TCG and TCA codons. Consequently, genes from the mobile genetic element using these codons are stochastically mis-synthesized. Genes in the host genome do not use TCG and TCA codons (pink) and are therefore unaffected by ambiguous decoding. **b**. A mobile genetic element encoding its own tRNAs invades a cell with a refactored genetic code. Competition between host and invading tRNAs leads to ambiguous decoding of codons. Consequently, genes from the mobile genetic element using these codons are stochastically mis-synthesized. If the refactored code is used to encode for essential host genes, these will also be stochastically mis synthesized. This will lead to the death of the host cell and provides an additional layer of defense. **c**. T4-like phage encoding a seryl-tRNA (tRNA_UGA_^Ser^) successfully infect Syn61Δ3 but not cells with a refactored genetic code. Plaque count indicates the number of successfully replicating phage obtained from infection with 1.1 × 10^10^ plaque-forming units (PFU)/mL (phage 12) and 7.5 × 10^9^ PFU/mL (phage 6). Independent replicates shown as individual dots (*n* = 3, bars indicate standard deviation). **d**. T4-like phage encoding a seryl-tRNA (tRNA_UGA_^Ser^) successfully infect Syn61Δ3 but not cells with a refactored and locked genetic code. Plaque count indicates the number of successfully replicating phage obtained from infection with 1.1 × 10^10^ plaque-forming units (PFU)/mL (phage 12) and 7.5 × 10^9^ PFU/mL (phage 6). Cells contain a cognate spectinomycin resistance gene rendering the refactored genetic code essential in the presence of spectinomycin; all experiments were performed in the presence of spectinomycin. Independent replicates shown as individual dots (*n* = 3, bars indicate standard deviation). Data is reproduced from prior work.^8^

We previously showed that conjugative transfer of an F plasmid encoding its own tRNA [F (WT + serT)] was reduced, but not eliminated, by genetic code refactoring.^8^ By writing an essential gene in the target cell according to the refactored genetic code we made the refactored code essential, we call this a locked-in refactored code. In a cell with a locked-in refactored code the import of a seryl-tRNA leads to stochastic amino acid misincorporation in essential host proteins that are encoded in essential genes written in the refactored code (Figure 2b). We previously showed that in cells with a refactored and locked-in genetic code conjugative transfer of F (WT + serT) was ablated.^8^

Further, we previously identified phage from the natural environment that encode for a seryl-tRNA (tRNA^Ser^_UGA_) on their genome and showed that such phage can infect Syn61Δ3 (Figures 2 and S7). We also showed that we could ablate plaque formation by these phage through code-locking (Figure 2c).^8^ Here, we assayed plaque formation of phage 6 and 12 on cells with a refactored, but not locked, genetic code and observe that genetic code refactoring alone is sufficient to ablate plaque formation (Figure 2d). Furthermore, we show that ablation of plaque formation correlates with the inability of phage to propagate in cells over 24 h in liquid culture (Figure S8), consistent with results from others.^9^

We conclude that there is a striking difference between conjugative transfer, where code-locking is essential for ablation, and phage infection, where genetic code refactoring without code-locking is sufficient for resistance.

Code refactoring is sufficient to inhibit plaque formation.

### Code-Locking Enables Stable Phage Resistance

We realized that while code-locking might not be necessary for short-term-phage resistance it could be essential for organisms to maintain robust resistance to horizontal gene transfer and phage infection over time. The tRNAs responsible for code refactoring can be inactivated through a variety of mechanisms, such as mutation, deletion, or silencing. This inactivation would essentially revert the cell with a refactored genetic code back to codon compressed cell with decoder deletion (like Syn61Δ3) and render it susceptible to infection by phage that carry a suitable tRNA gene (Figure 3a). If the code is locked, however, the tRNAs responsible for refactoring are essential and cannot be inactivated without killing the cell. We hypothesized that this essentiality would ensure the robust maintenance of resistance to horizontal gene transfer and phage infection in organisms with locked codes (Figure 3b).

**Figure 3:**
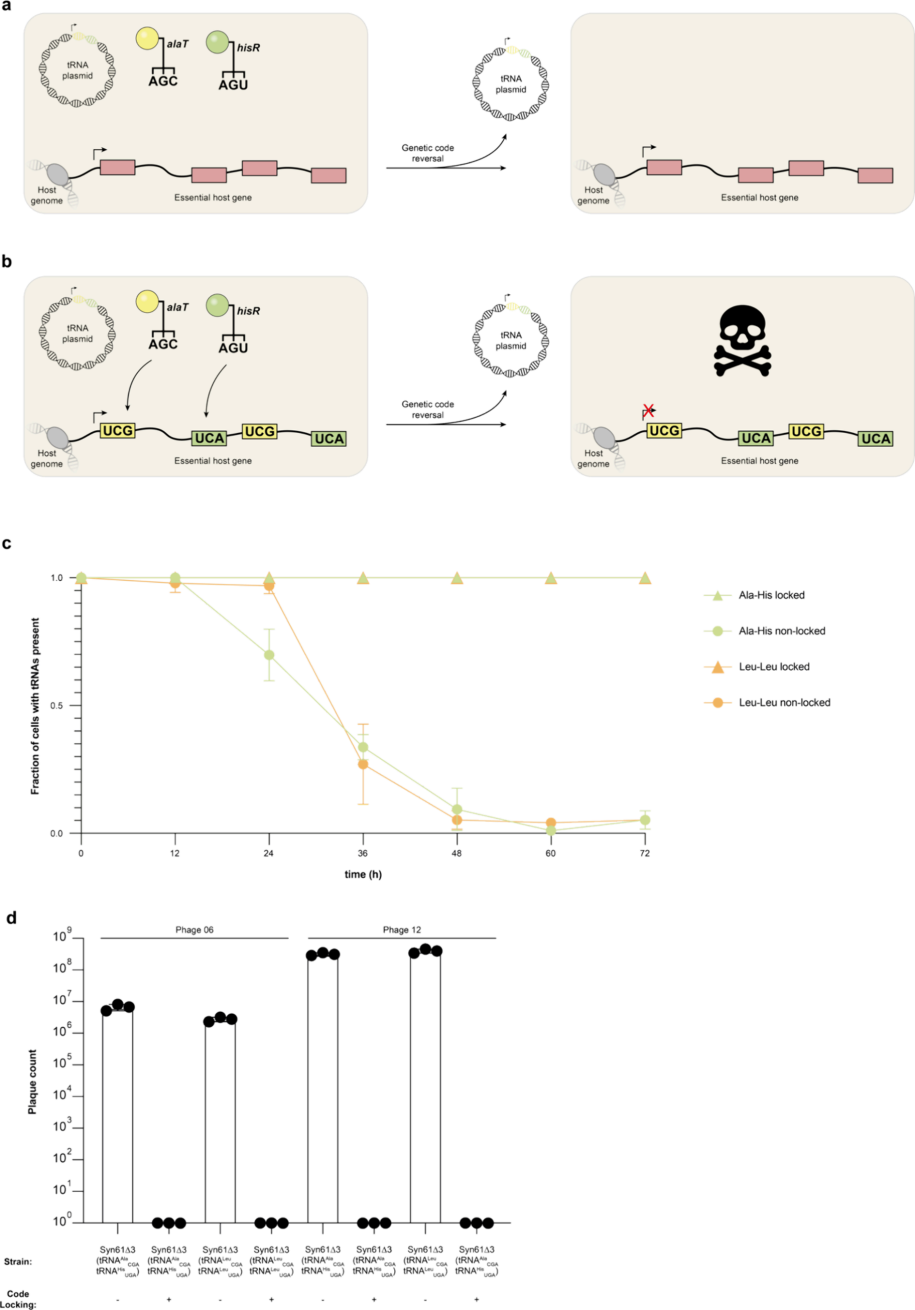
Code-locking ensures stability of refactored genetic codes. **a**. In cells with a refactored genetic code where this code is not locked, the expression of essential genes does not depend on the presence of engineered tRNAs for code refactoring. If the genetic code is reversed (tRNAs are lost or deactivated) cells survive. Therefore, refactored genetic codes are not stable in populations of cells without code-locking. **b**. In cells with a refactored genetic code where this code is locked, the expression of essential genes depends on the presence of engineered tRNAs for code refactoring. If the genetic code is reversed (tRNAs are lost or deactivated) cells die. Therefore, refactored genetic codes are stable in populations of cells with code-locking. **c**. Cells were passaged every 12 h and assessed for the presence of the refactored code. Cells with code-locking retained the genetic code in all cases, demonstrating stability of code refactoring. In contrast, cells without code-locking lost the refactored code. Cells either contain a codon compressed SpecR resistance gene (not locked: −) or a cognate SpecR gene (SpecR TCG-Ala, TCA-His/SpecR TCG-Leu, TCA-Leu) (locked: +); all experiments were performed in the presence of spectinomycin. Dots show the mean of three replicates and bars indicate the standard deviation. **d**. Phage encoding a seryl-tRNA_UGA_ infect cells with unstable genetic codes, but not cells with stably refactored codes. Cells from the time course (a) with and without code-locking were subject to infection with T4-like phages (Phage 06/12). Plaque count indicates the number of successfully replicating phage obtained from infection with ∼5 × 10^8^ PFU (Phage 12) and ∼1 × 10^7^ PFU (Phage 6). Cells either contain a codon compressed SpecR resistance or a cognate SpecR gene (as in b); all experiments were performed in the presence of spectinomycin. Independent replicates shown as individual dots (*n* = 3, bars indicate standard deviation).

We modeled the stability of alternative genetic codes in the presence and absence of code-locking. tRNAs responsible for the alternative decoding of TCG and TCA codons were encoded on a low-copy plasmid. A second plasmid encoded for a variant of a spectinomycin resistance gene (*Spec* ^*R*^); For cells without code-locking: *Spec* ^*R*^(Δ*TCG*, Δ*TCA*), for cells with code-locking: *Spec* ^*R*^ *(TCG: Ala, TCA: His)* and *Spec* ^*R*^ *(TCG: Leu, TCA: Leu)* respectively. Cells were serially passaged in the presence of spectinomycin (with no direct pressure to maintain the tRNA plasmid). In each passage we measured the fraction of cells that maintained the plasmid encoding the tRNAs. We find that in populations of code-locked cells the tRNA plasmid is maintained in all cells, while in populations of cells without code-locking the tRNA plasmid is lost from an increasing fraction of cells over time. We conclude that code-locking stabilizes alternative codes and acts to maintain code refactoring (Figure 3c).

We took cells with a refactored code and cells with a locked code from the sixth passage of the previous experiment and exposed each population to phage 12 and 06. We observed that code-locked populations retained resistance to phage infection, while populations that were not code-locked were susceptible to phage infection (Figure 3d). This demonstrated that cells with refactored genetic codes lose resistance within a few days if they are not code-locked, and that code-locking is essential for cells to retain resistance to phage over time.

## Discussion

In this study, we expanded the set of accessible refactored genetic codes from 16 to 60. Although the efficiency of these additional genetic codes is limited, this work may serve as a starting point for the creation of number of distinct genetically isolated bacterial populations.^8^ Genetic codes that use the same codon to encode amino acids with different chemical properties could be used to enhance genetic isolation, since the corresponding substitutions may be less likely to produce functional proteins.^21^ Lastly, efforts to expand the set of refactored genetic codes could enable creation of genetic codes with more suitable properties for protein evolution.^22,23^

In principle, the reassignment of TCG and TCA to 19 distinct amino acids should be possible, enabling the creation of 361 unique refactored genetic codes. However, many of the anticodon modified tRNAs that we tested were not functional and some of them led to reduced decoding efficiency. Many factors could be responsible for this: 1) tRNA misfolding could lead to a nonfunctional tRNA. 2) Disruption of the interaction of the tRNA with its cognate amino-acyl tRNA synthetase (aaRS) or an unfavorable interaction with a different noncognate aaRS could lead to a lack of acylation or misacylation. 3) Mutation of the anticodon might disrupt or alter post-transcriptional modifications of the tRNA, especially those that occur in the anticodon stem-loop. Post-transcriptional modifications are involved in maturation, folding, cellular stability, amino-acylation, and translation and their disruption might affect the tRNAs functionality at any of these levels.^24^ (Figure S9).

All these effects limit codon reassignments achievable through simple modification of tRNAs alone. Efforts to reassign codons to a wider-range of amino acids and to improve incorporation efficiency may need to further characterize the tRNAs with altered codons to assess their stability, aminoacylation and post-transcriptional modifications. This data could then inform directed evolution efforts aimed at engineering tRNAs, aaRSs and possibly tRNA modifying enzymes. The repurposing of orthogonal tRNA/aaRS pairs from distant species offers another avenue for further codon reassignment in .^25,26^ In the future, engineering of further refactored genetic codes will be advanced through the compression of more codons in the canonical genetic code as we recently demonstrated through the creation of Syn57 that removed 7 codons from the genetic code; Syn57 will enable the reassignment of up to 7 codons to a wide range of natural and unnatural amino acids.^41^

We have previously shown that for simple genetic elements, such as conjugative plasmids, refactoring the structure of the genetic code alone is not sufficient to render cells completely resistant to their transfer; the refactored genetic code needs to be locked into the synthetic host cell. In contrast we now show, complete resistance to more complex genetic systems, such as phage, can be achieved by refactoring the genetic code without code-locking.

Several factors may contribute to how different genetic elements interact with cells possessing a refactored code, including: (1) the number of target codons in the transferred genetic element, (2) the complexity of the life cycle of the transferred genetic element and, (3) the importance of multimeric protein assemblies in the life cycle of the element.

We analyzed the genomes of phage 6, phage 12 and the RK2 F plasmid for the presence of target codons (TCG and TCA) in the predicted ORFs. We observe that the number of target codons in the phage investigated here is more than three times larger than in the RK2 F plasmid (Figure 4a). This may be a contributing factor to the difference we observe. As more positions are affected by amino acid misincorporation, the chance that there is a deleterious effect is potentially higher. Additionally, we observe that the phage genomes show an about 25% increased frequency of target codons in their genome in comparison to the F plasmid (Figure 4b). This may also contribute to the difference we observe in our experiments. With increased frequency of amino acid misincorporation the chance that an open reading frame is translated to make a nonfunctional protein is higher. While the relative usage of TCG and TCA codons in the genomes of phage 12, 6 and F′ (WT *+serT*) could in principle contribute to the observed difference we deem this to be unlikely, because reassigning both these codons to the same amino acid (e.g., leucine) still leads to a difference between phage infection and conjugative transfer^8^ (Figure 2).

**Figure 4:**
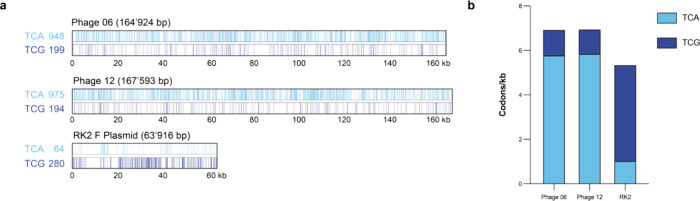
Genomic analysis of mobile genetic elements. **a**. Comparative genome analysis of T-4 like phages (Phage 06 and Phage 12) and the RK2 F plasmid. On the *x*-axis the size is indicated in kilobases. Positions where a TCA codon occurs are marked with a vertical, light blue line (top). Positions where a TCG codon occurs are marked with a vertical, dark blue line (bottom). **b**. Codon usage in mobile genetic elements. On the *x*-axis the total frequency of target codons (TCA and TCG) is indicated. TCA codon frequency is represented in light blue, TCG codon frequency in dark blue.

Differences in the mechanism of conjugative transfer and phage infection may also account for the difference we observe in our experiments. For successful phage infection and plaque formation the whole life cycle of the phage needs to be completed (Figure S10).^27,28^ This is a highly complex process that requires tight temporal regulation. For T4-like phages, such as phage 12 and 6, at least 62 phage encoded genes are essential for completion of the whole life cycle.^29^ In contrast conjugative transfer and subsequent colony formation is a much simpler process. All proteins involved in plasmid transfer and recircularization are either expressed from the recipient cell genome or transferred from the donor alongside the DNA.^30^ Subsequently, solely the replication of the plasmid and its proper segregation during cell division need to be ensured for successful colony formation (Figure S10). This process requires very few genes from the conjugative element to be functionally expressed.^30^

Furthermore, phage proteins form complex interactions that may be susceptible to dominant negative effects. Some amino acid substitutions in structural proteins of viruses are known to show dominant negative phenotypes.^31,32^ Mutation in structural proteins may disrupt processing, or oligomerization and therefore can be detrimental to correct particle assembly. In T4-like phages the major capsid protein (gp23) forms hexamers that are the basis for particle assembly.^33^ In the gp23 of phages 06 and 12 there are three surface exposed serine residues that are encoded by TCA (Figure S11). Amino acid misincorporation at one of these positions in a subset of gp23 could disrupt capsid assembly and thereby have a dominant negative effect on plaque formation.

We expect resistance to phage infection through refactored genetic codes to generalize across most if not all families of phage. Sense codon compression and decoder deletion has been demonstrated to make Syn61Δ3 resistant to phage from distant evolutionary families (Supplementary Table 1).^7^ Interestingly, phage encoding an appropriate seryl-tRNA–that have been shown to infect Syn61Δ3–cover a much smaller diversity. Phage 12 and phage 06 are T4-like phages belonging to the tequatrovirus genus, and four of the 12 phages reported to infect Syn61Δ3 in another study^8,9^ also display a high degree of genomic identity to T4 (Supplementary Table 1). The remaining eight phages, capable of infecting Syn61Δ3, in the other study were from the felixounavirus genus (Supplementary Table 1).^9^ Phage from other families that can infect Syn61Δ3 have not been discovered yet. Strikingly, genetic code refactoring is sufficient for resistance to all phages described to infect Syn61Δ3.^8,9^

One potential mechanism to escape resistance by genetic code refactoring involves the evolutionary loss or avoidance of specific codons by phages. For instance, a certain RNA virus that infects CTG yeast—a strain of yeast that deviates from the canonical genetic code in its decoding of the CTG codon—has eliminated all but one CTG codon from its genome, rendering its genes interpretable by the host’s translational machinery.^34^ Presumably, minimal usage of TCR codons could enable a phage to infect Syn61Δ3 cells with a refactored genetic code. However, adapting to a new codon landscape is not trivial—in natural coevolution, viruses and their hosts gradually shift genetic codes over time. Engineered recoded hosts are recoded in a laboratory setting and very rapidly on an-evolutionary time scale, without opportunity for the phage to gradually coadapt. The phages we investigated have over 1000 TCR codons in their genome and would therefore require many simultaneous mutations in order to overcome genetic code refactoring. Other mechanisms of escape may target the host translational machinery to revert genetic code refactoring, for example through targeted degradation of engineered tRNAs. Phage adaptation poses a risk to the functionality of systems for genetic isolation outside the laboratory over long time scales. In the future, we may experimentally test evolutionary phage adaptation and identify mechanisms of phage adaptation by performing coevolution experiments where we coculture organisms with refactored genetic codes and phages. Further, the risk of phage adaptation can be reduced by more drastic refactoring of the genetic code – the farther the safer.^35^ We have recently compressed more codons in to generate strains with genetic codes that deviate more radically from the canonical genetic code.^41^

Others have claimed complete resistance of cells with refactored genetic codes to infection by phage carrying their own tRNAs.^9^ Our results confirm that code-locking is not essential for short-term resistance to T4-like phage infection. However, we show that code-locking is essential to maintain this resistance over time. In our experiments, cells without code-locking rapidly reverse code refactoring and consequently become susceptible to infection by this subset of viruses. While different mechanisms of reversal (e.g.: mutations, silencing, and deletion of tRNAs) may operate on different time scales, our results suggest that code refactoring without code locking may not generate organisms that are suitable for applications outside a research laboratory, where infections with viruses carrying their own tRNAs may occur sporadically and at unpredictable intervals.

## Conclusions

Bioproduction facilities are key for the production of many pharmaceuticals, enzymes and food additives.^36–38^ The resilience of bioproduction plants is critical for their stable integration into the economy. Sporadic and unpredictable invasion of cells used in bioproduction by mobile genetic elements, including viruses, can cause financial losses and more importantly generate unpredictability and disruption in vital supply chains.^11,39^ Synthetic organisms with refactored genetic codes and essential genes that lock the refactored code are resistant to mobile genetic elements (including those carrying their own translation factors) written in the canonical genetic code. Locking the synthetic code into the organism provides a cell-autonomous mechanism to ensure temporal stability of the resistance phenotype; we anticipate that this will be critical for enabling organisms to maintain resistance to repeated and sporadic infection. In industrial settings this may contribute to greater stability in biomanufacturing-based supply chains.

Due to the universality of the genetic code, locked genetic codes can in principle be applied to any organism. In practice, advancements in methodology for genome synthesis may rapidly enable refactoring of the genetic code of many organisms used in research and industry.^40^

## Supplementary Material













## Data Availability

The accession numbers for the sequencing data described in the text are provided in Supplementary Table 4. Phage genome sequences were uploaded to Genbank accession numbers can be found in Supplementary Table 4. The authors agree to provide any materials and strains used in this study upon request. Numerical values for all graphs are available in Supplementary Table 3.

## Notes

Phage phylogenetic classification (XLSX)
Triage of functional tRNAs based on MS data (XLSX)
Raw data for bar graphs in main and supplementary figures (XLSX)
Description of constructs, phages, and sequencing data used in this study (XLSX)
Containing maps of plasmids used in this study (ZIP)
Screen of anticodon modified E. coli tRNAs; mass spectrometry tRNAs with CGA anticodon; mass spectrometry tRNAs with UGA anticodon; on- and off-target activity of anticodon modified tRNAs; identification of tRNAs suitable for genetic code refactoring; translational activity of evolved and viral leucyl-tRNAs; phage identification and annotation; 24 h phage propagation assay; possible effect of anticodon modification on tRNA function; phage replication is a more complex biological function than conjugation; and structure of major capsid protein gp23 (PDF)
