# Supplementary material for: Genetic Code-Locking Confers Stable Virus Resistance to a Recoded Organism
Source: Biochemistry. 2025 Jul 1;64(14):3093–103. doi: 10.1021/acs.biochem.5c00075 (PMC12269064; doi:10.1021/acs.biochem.5c00075)
Supplement: Supplementary file 6 [file bi5c00075_si_006.pdf]

Supporting Information: Genetic code-locking confers stable virus resistance to a recoded organism

Jérôme F. Zürcher[1,†,*], Alexandre Dickson[1,†], Tomás Kappes[2], Askar A. Kleefeldt[1], Kim C. Liu[1], George P. C. Salmond[2] & Jason W. Chin[1,*]

[1]Medical Research Council Laboratory of Molecular Biology, Francis Crick Avenue, Cambridge, CB2 0QH, England, UK

[2]Department of Biochemistry, University of Cambridge, Tennis Court Road, Cambridge, CB2 1GA, England, UK.

[†]These authors contributed equally to this work.

[*]Correspondence: jzurcher@mrc-lmb.cam.ac.uk, chin@mrc-lmb.cam.ac.uk

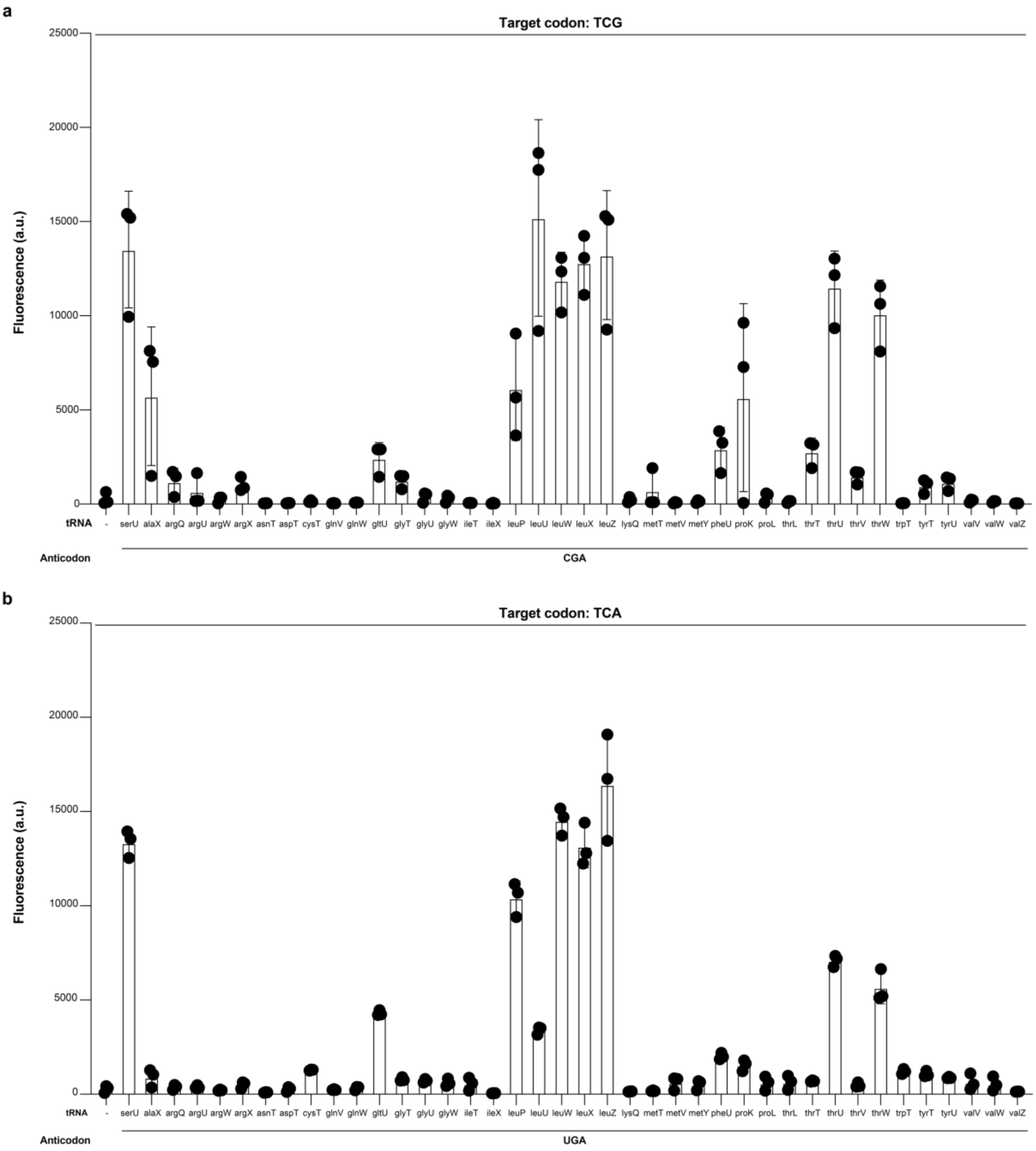

Figure S1 | Screen of anticodon modified *E. coli* tRNAs

Anticodon (CGA or UGA) modified versions of all endogenous *E. coli* tRNAs (with exception for seryl-tRNAs and previously reported *alaT*, *hisR*, *leuQ*, and *proM*) were tested for their translational activity on target codons (TCG or TCA). Individual measurements (n=3), mean, and standard deviation are indicated.

a, The anticodon of endogenous *E. coli* tRNAs was modified to CGA. The translational activity of these tRNAs was assessed through suppression of a TCG codon at position 3 of a sfGFP

gene. Measured fluorescence serves as a read-out for translational activity of a given tRNA on a TCG codon. Cells where no tRNA was expressed (-) and cells that express *serU* endogenous seryl-tRNA serve as negative and positive controls respectively.

b, The anticodon of endogenous *E. coli* tRNAs was modified to UGA. The translational activity of these tRNAs was assessed through suppression of a TCA codon at position 3 of a sfGFP gene. Measured fluorescence serves as a read-out for translational activity of a given tRNA on a TCA codon. Cells where no tRNA was expressed (-) and cells that express *serU* endogenous seryl-tRNA serve as negative and positive controls respectively.

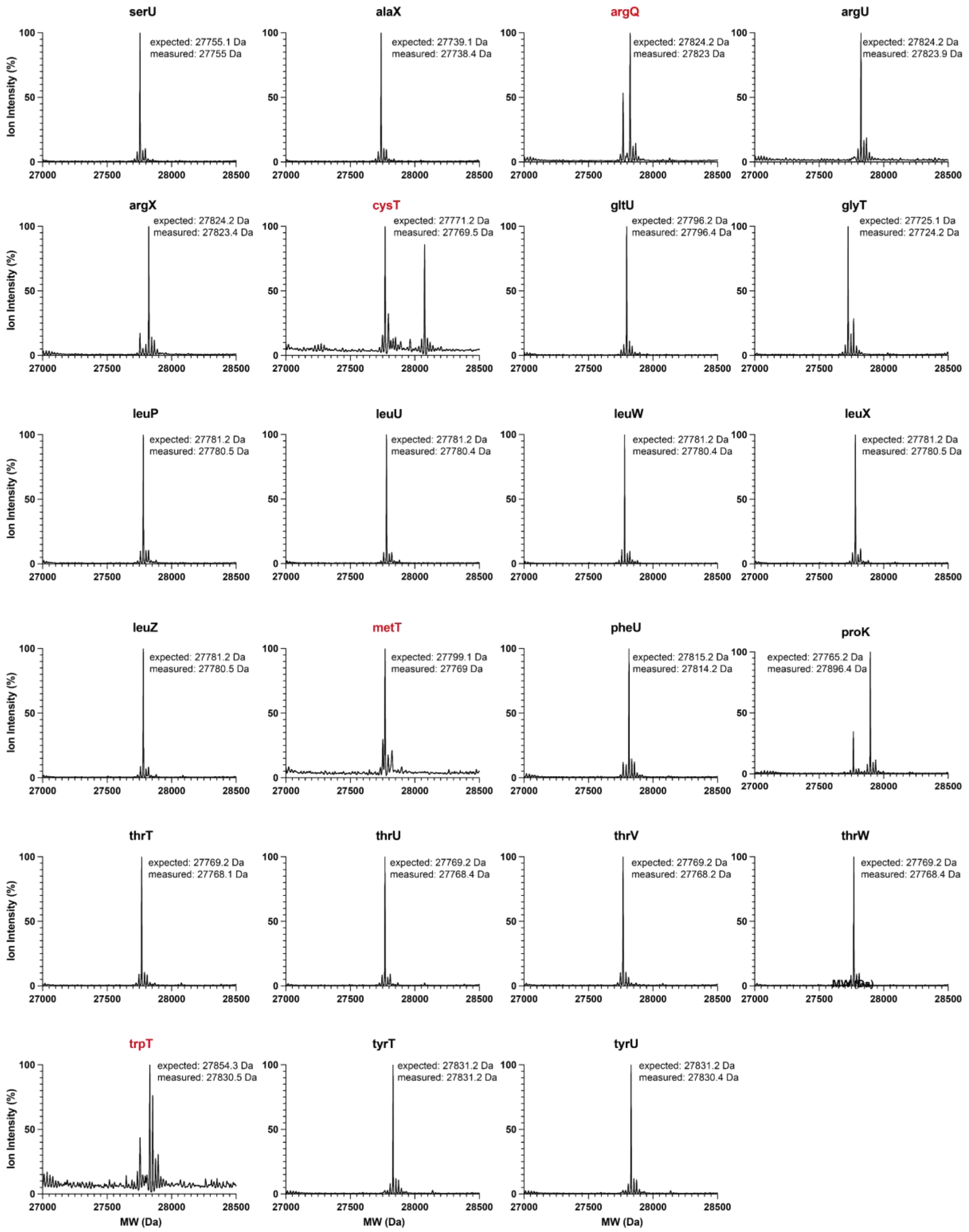

Figure S2 | Mass spectrometry tRNAs with CGA anticodon

The identity of the amino acid incorporated at TCG codons was determined by electrospray ionization mass spectrometry (ESI-MS) of purified sfGFP which was expressed from a gene

with a TCG codon at position 3 in the presence of a tRNA with a CGA anticodon. *serU* encoding for $tRNA^{Ser}_{CGA}$ was used as a control. Expected and measured masses (for the main peak) are indicated. For a full list of expected and found masses and incorporation specificities see **Supplementary table 2**.

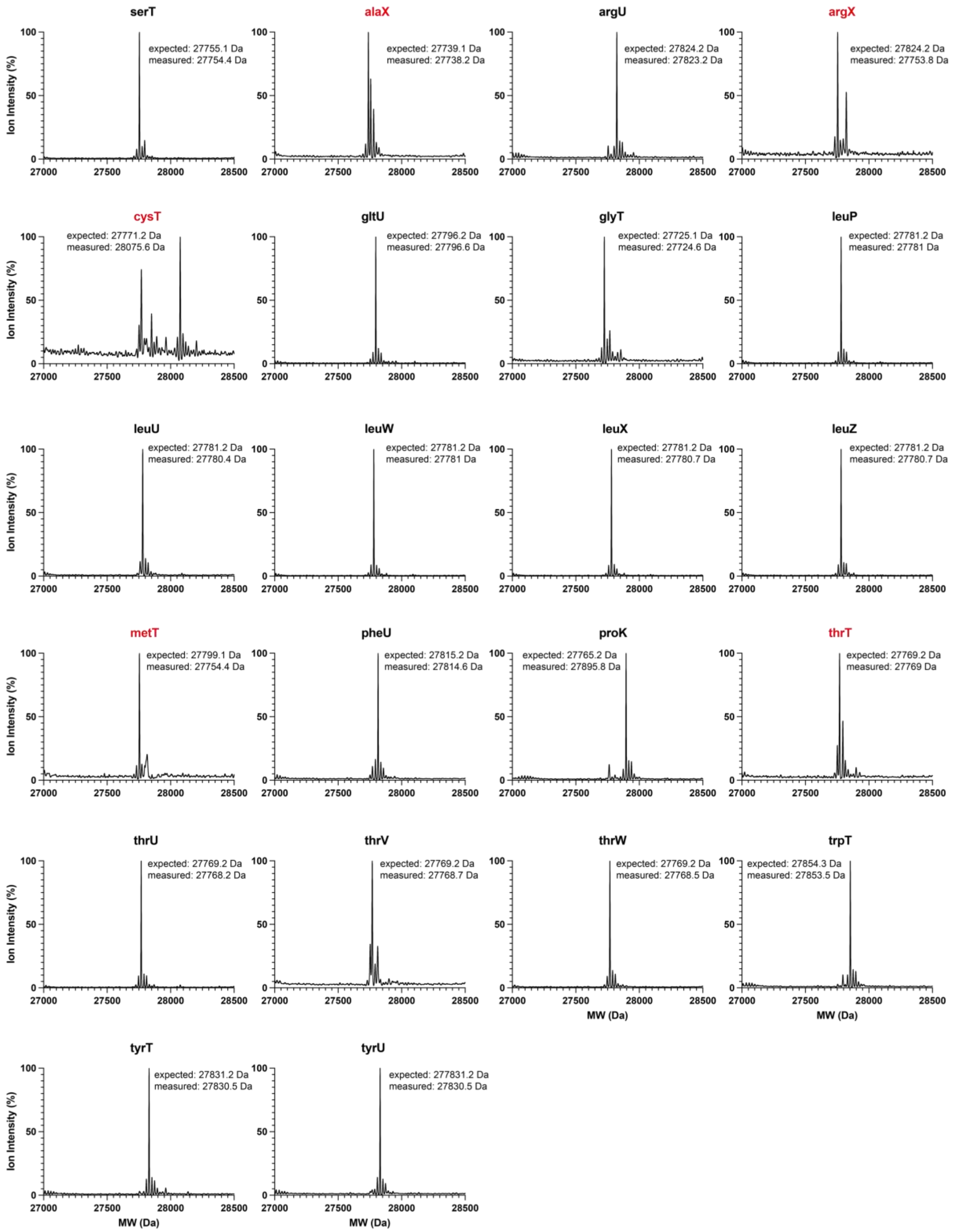

Figure S3 | Mass spectrometry tRNAs with UGA anticodon

The identity of the amino acid incorporated at TCA codons was determined by electrospray ionization mass spectrometry (ESI-MS) of purified sfGFP which was expressed from a gene

with a TCA codon at position 3 in the presence of a tRNA with a TGA anticodon. *serT* encoding for $tRNA^{Ser}_{UGA}$ was used as a control. Expected and measured masses (for the main peak) are indicated. For a full list of expected and found masses and incorporation specificities see **Supplementary table 2**.

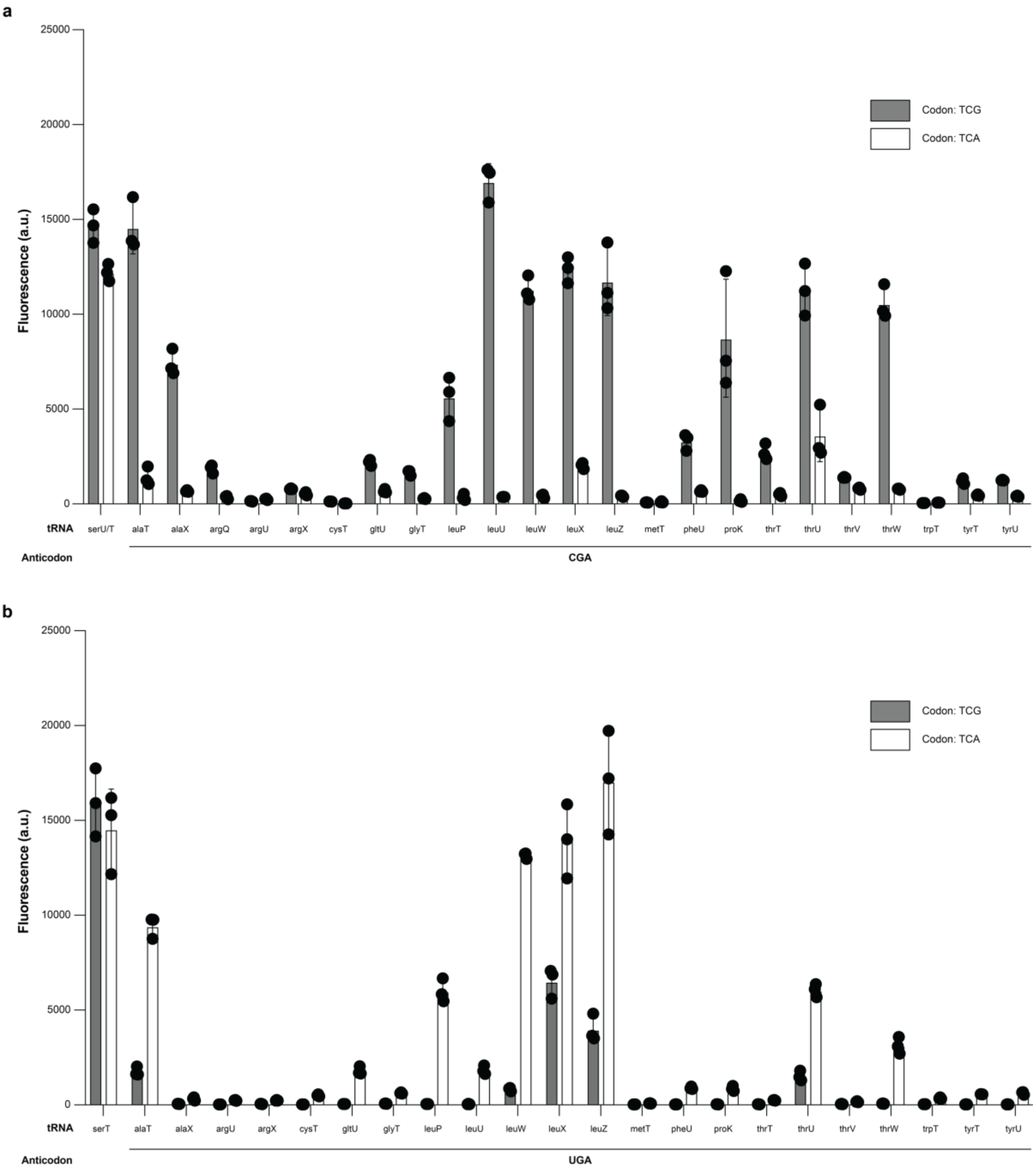

Figure S4 | On- and off-target activity of anticodon modified tRNAs

a, A subset of endogenous *E. coli* tRNAs with anticodon modified to CGA was assessed for their translational activity on TCG (target) and TCA (off-target) codons through codon suppression at position 3 of a sfGFP gene. Measured fluorescence serves as a read-out for translational activity of a given tRNA on a given codon. Individual measurements (n=3), mean, and standard deviation are indicated.

b, A subset of endogenous *E. coli* tRNAs with anticodon modified to UGA was assessed for their translational activity on TCA (target) and TCG (off-target) codons through codon suppression at position 3 of a sfGFP gene. Measured fluorescence serves as a read-out for translational activity of a given tRNA on a given codon. Individual measurements (n=3), mean, and standard deviation are indicated.

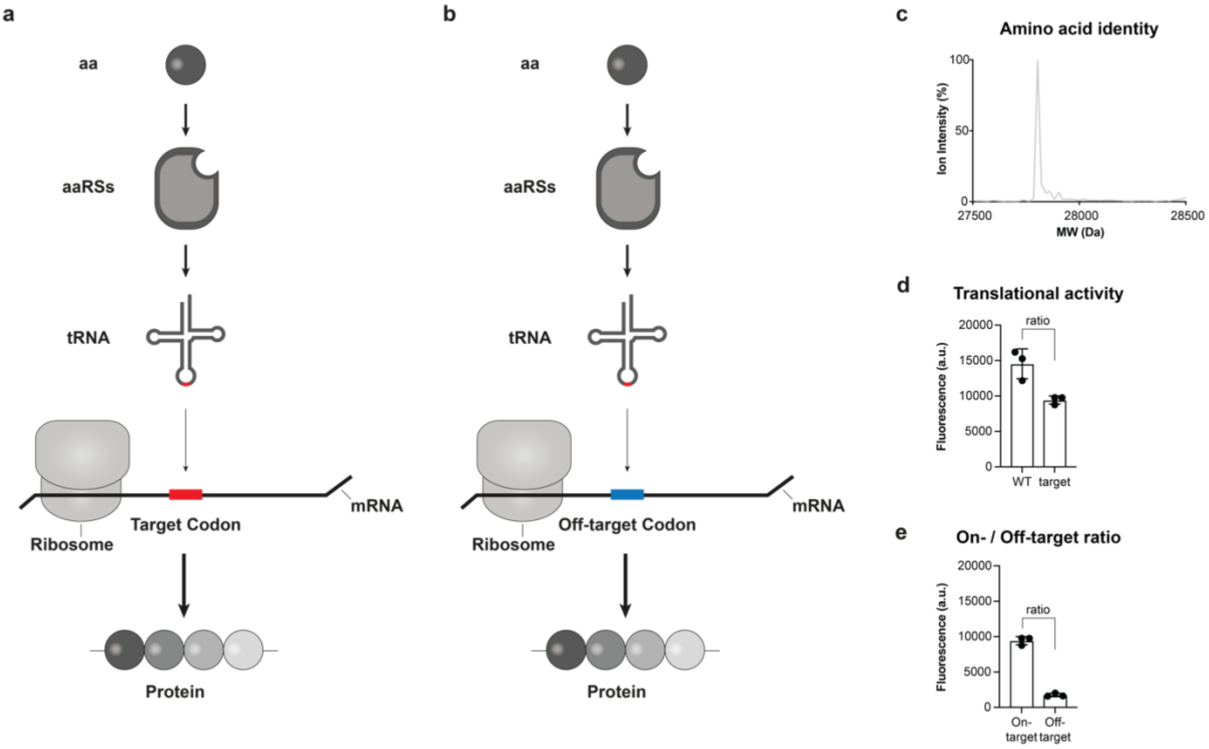

Figure S5 | Identification of tRNAs suitable for genetic code refactoring

a, The activity of an endogenous *E. coli* tRNA with a modified anticodon (CGA or UGA) is assessed on its target codon (TCG or TCA respectively).

b, The activity of an endogenous *E. coli* tRNA with a modified anticodon (CGA or UGA) is assessed on its off-target codon (TCA or TCG respectively).

c, For tRNAs with translational activity on target codons the identity of the incorporated amino acid is assessed by mass-spectrometry. Only tRNAs that specifically incorporate their cognate amino acid are suitable for genetic code refactoring.

d, The activity of modified tRNAs on target codons is compared to the activity of WT tRNAs (serU and serT for TCG and TCA respectively). Only tRNAs that have substantial translational activity on target codons are suitable for genetic code refactoring.

e, The activity of modified tRNAs on target codons is compared to their activity on off-target codons. Only tRNAs with activity specific for target codons are suitable for genetic code refactoring.

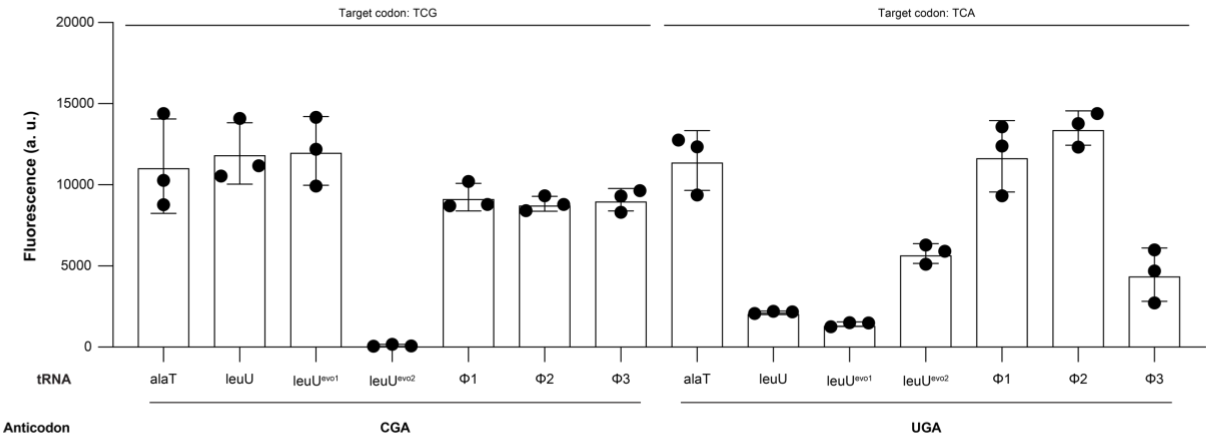

Figure S6 | Translational activity of evolved and viral leucyl-tRNAs

Phage derived and evolved tRNAs from another study[1] were assessed for their translational activity through codon suppression at position 3 of a sfGFP gene. Measured fluorescence serves as a read-out for translational activity of a given tRNA on a given codon. On the left, the activity of tRNAs with a CGA anticodon on TCG. On the right, the activity of tRNAs with a UGA anticodon on TCA. Experiment was performed with three replicates per measurement (n=3, individual values and standard deviation indicated). Evolved and viral leucyl-tRNAs have translational activity that is no higher than that of endogenous anticodon modified tRNAs.

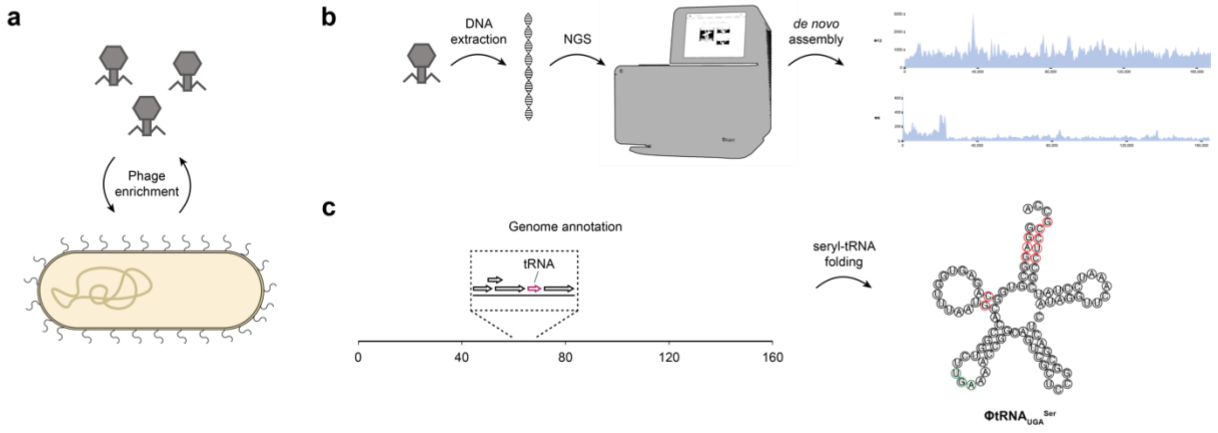

Figure S7 | Phage identification and annotation

a, Phage from environmental samples were enriched, determined to be able to infect Sny61Δ3 and clonally purified.

b, DNA was extracted from clonal phage particles and sequenced using next generation DNA sequencing (NGS). Phage genomes were *de novo* assembled from short-read sequencing data. Coverage at each position of the phage genome (x-axis) are shown on the y-axis. Data is reproduced from prior work[2].

c, *De novo* assembled phage genomes were annotated for both translated open reading frames and tRNAs. The seryl-tRNA identified from phage genomes were folded *in silico*. Seryl-tRNA identity elements crucial for recognition by the aaRS in *E. coli* are shown in red. tRNA anticodon is shown in green. Data is reproduced from prior work[2].

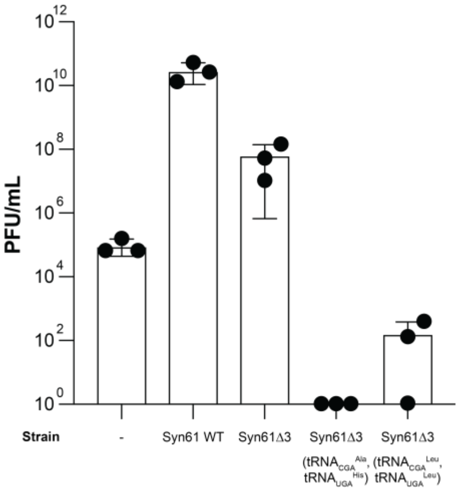

Figure S8 | 24 h phage propagation assay

Samples were inoculated with a defined titer of T4-like phage (Phage 12) (MOI = 0.001). After a 24 h growth, the titer of phage in each sample was assessed. In the control containing no cells the titter of phage remains unchanged after 24 h. In both Syn61 WT and Syn61Δ3 phage titer is increased by multiple orders of magnitude. In samples containing cells with refactored and locked genetic codes [Syn61Δ3 ($tRNA^{Ala}_{CGA}$, $tRNA^{His}_{UGA}$), Syn61Δ3 ($tRNA^{Leu}_{CGA}$, $tRNA^{Leu}_{UGA}$)] the titer of phage is decreased as compared to the control. Presumably because phage particles adsorb to such cells but are unable to replicate. Individual measurements (n=3), mean, and standard deviation are indicated.

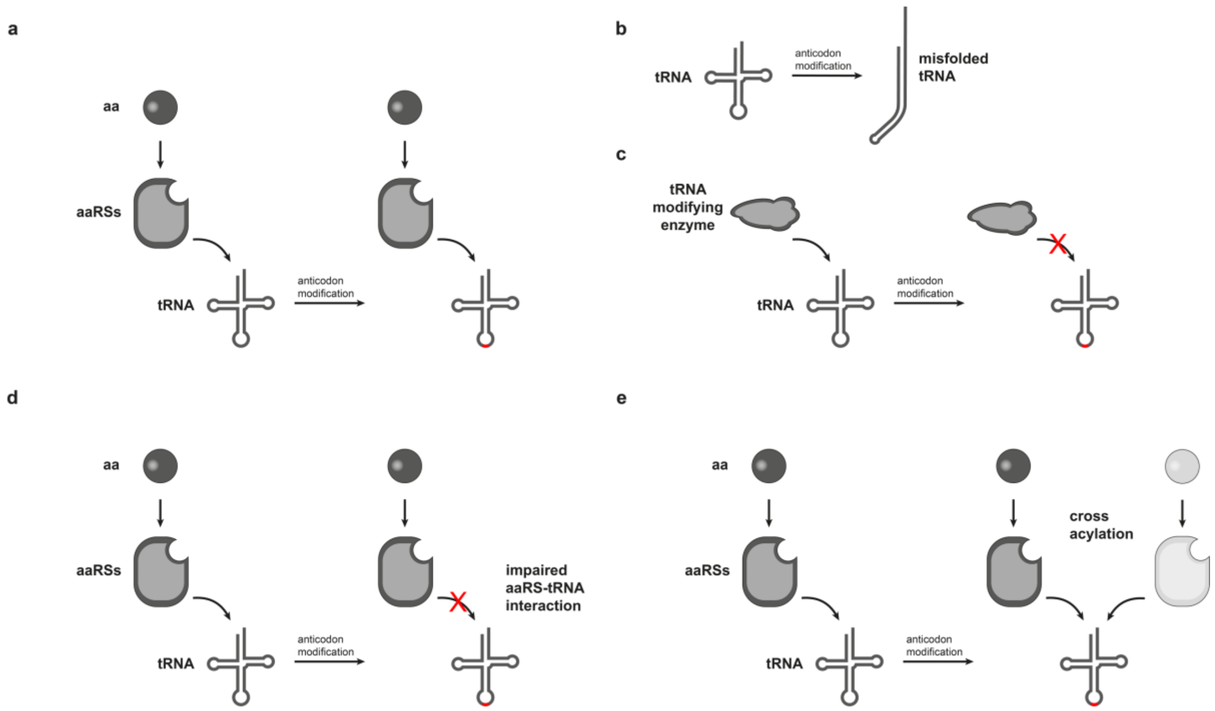

Figure S9 | Possible effect of anticodon modification on tRNA function

a, The anticodon of an endogenous *E. coli* tRNA is modified to CGA or UGA. The modified tRNA is structurally intact and still correctly recognised by its cognate amino acyl tRNA synthetase (aaRS). Therefore, this tRNA incorporates its cognate amino acid in response to TCG or TCA codons.

b, The anticodon of an endogenous *E. coli* tRNA is modified to CGA or UGA. The modified tRNA is misfolded and therefore has no translational activity.

c, The anticodon of an endogenous *E. coli* tRNA is modified to CGA or UGA. The modified tRNA is no longer post-transcriptionally edited, this can impact tRNA folding, cellular stability, amino-acylation, and translational activity.

d, The anticodon of an endogenous *E. coli* tRNA is modified to CGA or UGA. The modified tRNA is structurally intact but is no longer recognised by its cognate aaRS. Therefore, this tRNA has no translational activity.

e, The anticodon of an endogenous *E. coli* tRNA is modified to CGA or UGA. The modified tRNA is structurally intact but recognised by an aaRS that is different from its cognate aaRS. This leads to amino acid misincorporation in response to TCG or TCA codons.

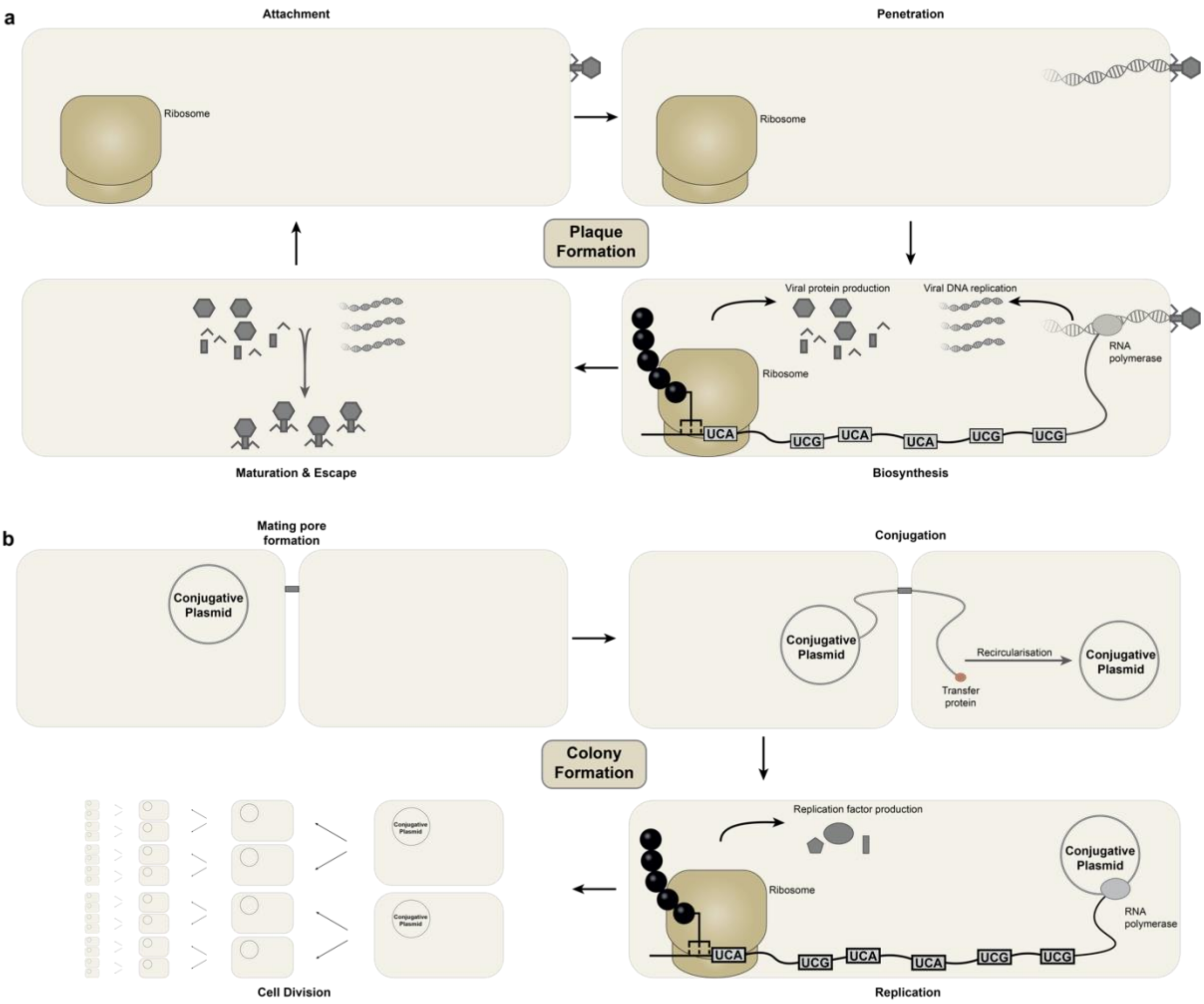

Figure S10 | Phage replication is a more complex biological function than conjugation. The formation of plaques from T-4 like phage infection is a more complex biological process than the formation of colonies after successful conjugative transfer.

a, For the formation of plaques a phage particle first needs to attach to a bacterial cell and inject its DNA into the cytosol. Subsequently, genes from the phage genome are transcribed and translated by the host machinery producing viral proteins. Moreover, the phage genome is replicated inside the cell. Finally, these components need to mature into fully functional phage particles that escape from the cell and infect neighbouring cells. This complex process requires many structural and regulatory proteins to be expressed correctly from the phage genome.

b, For the formation of colonies from recipient cells bearing a conjugative plasmid, the donor cell first needs to attach to the recipient. Subsequently, the F plasmid is transferred through the mating channel and recircularized inside the recipient cell. The process of attachment, transfer, and recircularization relies exclusively on proteins expressed in the donor cell. Then, proteins are expressed from the conjugative element enabling its replication and segregation during cell division. Division of the recipient cell bearing the transferred plasmid leads to colony formation without further conjugation events.

a b

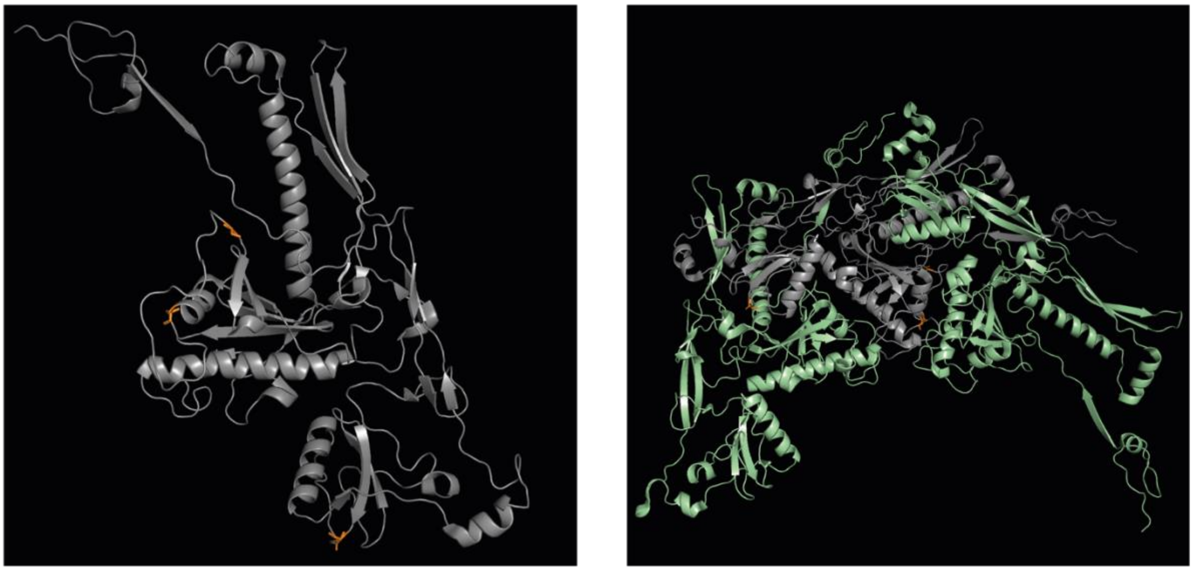

Figure S11 | Structure of major capsid protein gp23

a, Protein structure of major capsid protein gp23 from T4 phage. There are three serine residues encoded with TCA (highlighted in orange) and therefore subject to ambiguous decoding in cells with refactored genetic codes.

b, Binding interface of major capsid protein gp23; displayed are three subunits of gp23 that are part of a the hexameric capsid subunit. Serine residues encoded by TCA on the central subunit (in grey) are displayed in orange.

Supplementary table 1

This file contains the phylogenetic classification of phage that can infect Syn61Δ3 cells. It is provided as a separate file.

Supplementary table 2

This file contains the triage process of functional tRNAs for genetic code refactoring based on MS data. It is provided as a separate file.

Supplementary table 3: raw data for bar graphs in main and supplementary figures

This file contains the raw data underlying the bar graphs in main and supplementary figures. It is provided as a separate file.

Supplementary table 4

This file contains the description of constructs, phages, and sequencing data used in this study. It is provided as a separate file.

Supplementary file 1: zip file containing maps of plasmids used in this study

This file contains the maps of three plasmids used in this study in fasta format. All plasmids (as described in Supplementary table 4) are derivatives of these three plasmids. It is provided as a zip file.

Supplementary References

(1) Nyerges, A.; Vinke, S.; Flynn, R.; Owen, S. V.; Rand, E. A.; Budnik, B.; Keen, E.; Narasimhan, K.; Marchand, J. A.; Baas-Thomas, M.; Liu, M.; Chen, K.; Chiappino-Pepe, A.; Hu, F.; Baym, M.; Church, G. M. A Swapped Genetic Code Prevents Viral Infections and Gene Transfer. *Nat. 2023 6157953* **2023**, *615* (7953), 720–727. https://doi.org/10.1038/s41586-023-05824-z.

(2) Zürcher, J. F.; Robertson, W. E.; Kappes, T.; Petris, G.; Elliott, T. S.; Salmond, G. P. C.; Chin, J. W. Refactored Genetic Codes Enable Bidirectional Genetic Isolation. *Science* **2022**, *378* (6619), 516–523. https://doi.org/10.1126/science.add8943.
